# Supplementary material for: Mixed effects of a national protected area network on terrestrial and freshwater biodiversity
Source: Nat Commun. 2023 Sep 13;14:5426. doi: 10.1038/s41467-023-41073-4 (PMC10499833; doi:10.1038/s41467-023-41073-4)
Supplement: Supplementary file 1 — Supplmentary Information [file 41467_2023_41073_MOESM1_ESM.pdf]

Mixed effects of a national protected area network on terrestrial and freshwater biodiversity

Andrea Santangeli[1,2]*, Benjamin Weigel[1,3], Laura Antão[1], Elina, Kaarlejärvi[1], Maria Hällfors[1,4], Aleksi Lehikoinen[5], Andreas Lindén[6], Maija Salemaa[6], Tiina Tonteri[6], Päivi Merilä[6], Kristiina Vuorio[4], Otso Ovaskainen[1,7,8], Jarno Vanhatalo[1,9], Tomas Roslin[1,10,11], Marjo Saastamoinen[1,12]

Supplementary Tables:

Supplementary Table 1. Relationship between the protected area effect and species-level traits. The table shows impacts on species-level effect of protected area, modeled as a linear function of species traits. The response of the model included the species-specific effect of protection as estimated by the model presented in the main manuscript, Fig. 2, with values ranging from negative to positive. To account for taxon-specific differences, we fitted a separate model for each of the four taxonomic groups, assuming a Gaussian error distribution and an identity link. We included to a measure of body size (Size), which was log transformed for analysis, species thermal niche (STI – Species Temperature Index), and IUCN conservation status (two classes: threatened and Not threatened – the former set as the reference category). Note that not all traits were available for each of the four groups, see methods in the main text. Test statistics refers to the 2-tailed T-test (*lm* function in R) without adjustment for multiple testing.

**Birds:**	**Estimate**	**SD**	**t**	**p-value**
Intercept	0.004	0.001	2.782	0.006
Size	0.000	0.001	0.594	0.554
STI	0.000	0.001	-0.548	0.584
IUCN - Not Threatened	-0.001	0.002	-0.768	0.444
**Mammals:**				
Intercept	0.003	0.002	1.217	0.243
Size	0.000	0.001	-0.132	0.897
STI	0.000	0.001	-0.372	0.715
IUCN - Not Threatened	-0.003	0.003	-0.980	0.343
**Phytoplankton:**				
Intercept	-0.001	0.000	-2.066	0.040
Size	0.000	0.000	1.720	0.087
**Plants:**				
Intercept	-0.001	0.024	-0.028	0.978
Size	0.030	0.025	1.204	0.237
STI	-0.062	0.025	-2.498	0.018

Supplementary Table 2. Average value of protected buffer area for protected sites. The mean proportion of buffer area falling within a protected area, as defined for the sites classified as protected. We report the mean value per taxonomic group across all the sites (data used in the main analyses; 2nd column), as well as for the set of sites for which the first and second quartile (25% and 50% of the total sites with least cover) have been excluded (3rd and 4th columns), and which have been used for the sensitivity analyses (results shown in Supplementary Figure 2-4 below).

	Mean PA coverage		
**Taxa**	**All sites**	**75% sites with highest cover**	**50% sites with highest cover**
Birds	0.407	0.54	0.772
Mammals	0.0804	0.107	0.158
Plants	0.762	0.962	1
Phytoplankton	0.306	0.409	0.613

Supplementary Table 3. Model assessment and validation: Model convergence and fit statistics for the four full models (from each of the four taxa) for which results are shown in the main text. Statistics reported include the Potential Scale Reduction Factor (PSRF), the Area Under the Curve (AUC), Tjur R2 and the Root Mean Square Error (RMSE) for the birds, mammals, phytoplankton and plants models.

Taxa	PSRF	AUC	TjurR2	RMSE
Birds	1.002341	0.8459053	0.2155888	0.2487674
Mammals	1.002215	0.7683623	0.1124954	0.2268825
Phytoplankton	1.002079	0.80576022	0.09027419	0.2091302
Plants	1.001812	0.8572789	0.2412170	0.2814966

Supplementary Figures:

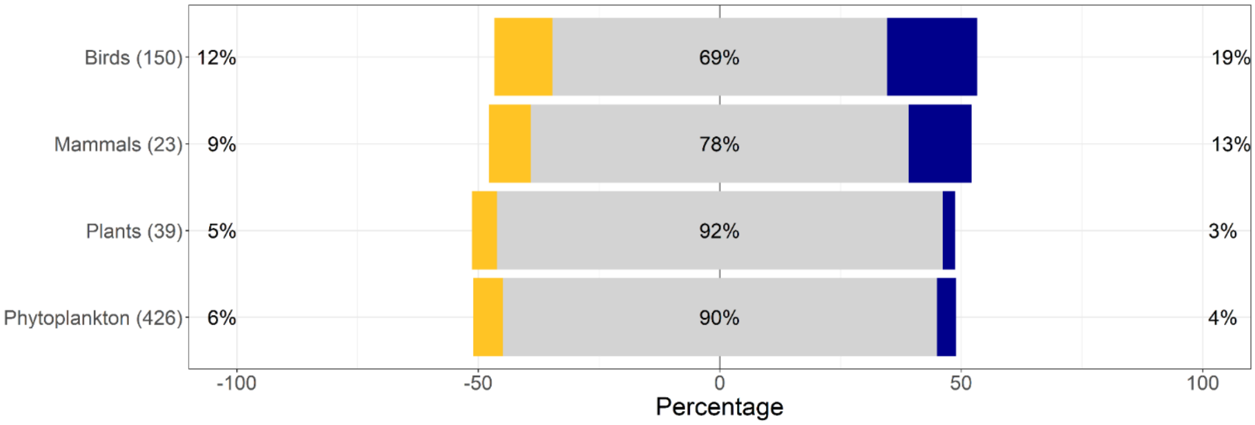

Supplementary Figure 1. Overall effectiveness of protected areas. Taxon-specific proportions of species with statistically supported positive (blue) or negative (yellow) versus no detectable effect (grey) of protected areas on their occurrence trends over time (using a 90% probability threshold to define a supported response). The total number of species for each taxon is given in brackets.

A. Results of analyses testing the effect of the size of protected area (Supplementary Figures 2-3)

To evaluate the effect of variation in the size of protected areas on biodiversity response, we repeated the main analyses on a subset including only half of the sites – corresponding to the protected areas with a size exceeding the median (within each taxon), and their corresponding unprotected site. The difference in the size (in km^2) of protected areas for this subset compared to that of all sites is very large for each of the four taxonomic groups: Birds: (mean PA size) all sites = 366 km^2, largest 50% sites = 720 km^2; Mammals: all sites = 111, largest 50% sites = 221; Plants: all sites = 878, largest 50% sites = 1741; Phytoplankton: all sites = 81, largest 50% sites = 161.

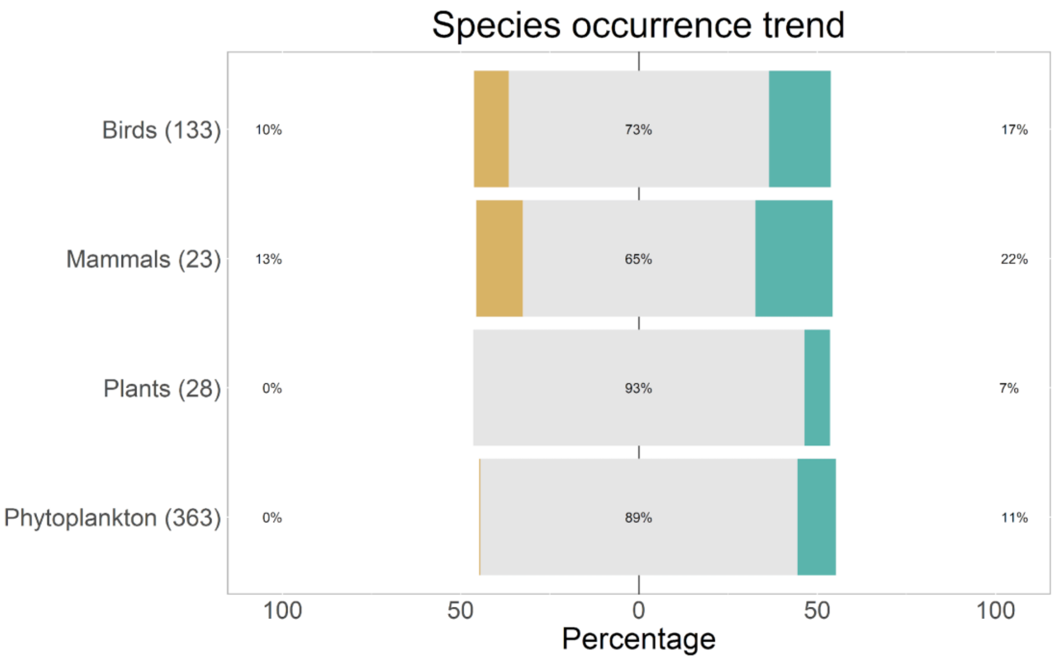

Supplementary Figure 2. Effect of variation in the size of protected areas on biodiversity response. The effectiveness of protected areas for the four taxonomic groups when only half of the sites - for which the protected area exceeded the median - were included, along with their corresponding unprotected sites. The figure shows the percentage of species with statistically significant support (defined with a 90% threshold) with a positive (green), negative (yellow), or no effect of protected areas on the occurrence trends of all species (total species number given within brackets).

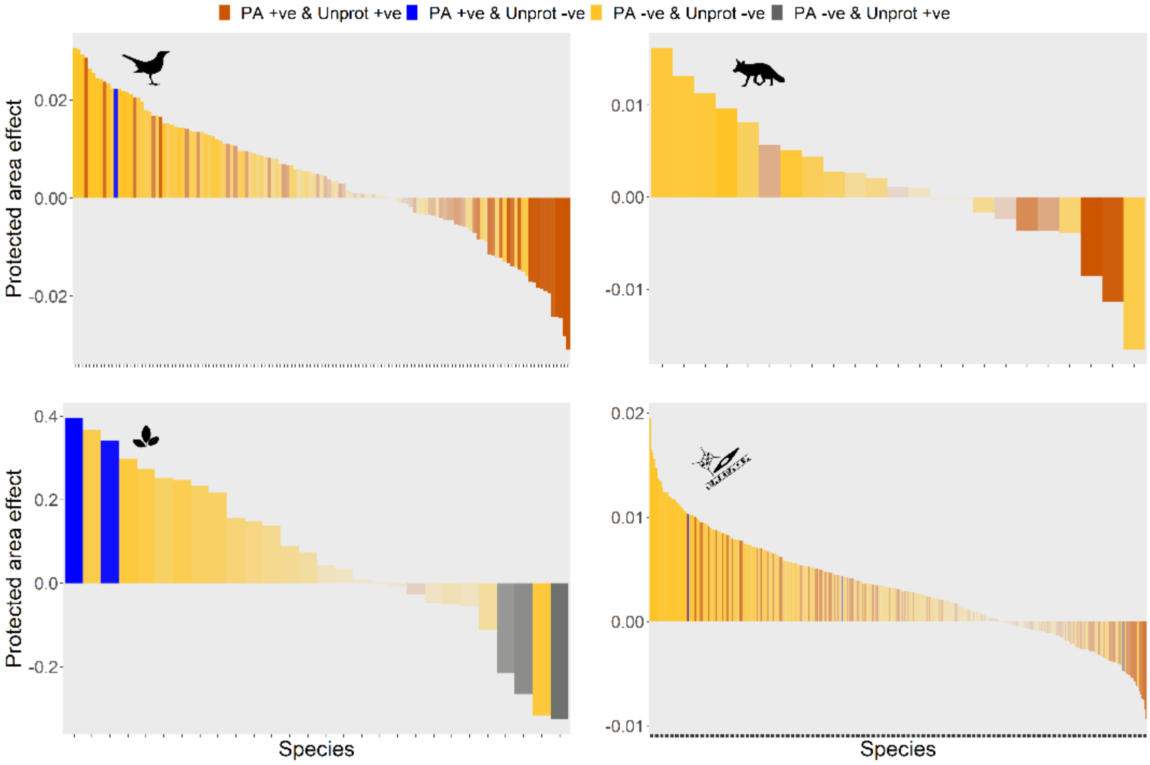

Supplementary Figure 3. Effect of variation in the size of protected areas on species-level response. The species-level effectiveness of protected areas for the four taxonomic groups when only half of the sites - for which the protected area exceeded the median - were included, along with their corresponding unprotected sites. Values are derived from the effect size of the interaction term between protection and year in the joint species distribution models (to be compared with the results using all sites in Fig. 3 of the main manuscript). Bars represent the effect of protected areas on a given species, from the most positive (left) to the most negative (right). Colours indicate the level of statistical support: the darker the colour the higher is the statistical support for the effect on the species. Bar colors represent classes of species occurrence trends within and outside of protected areas: Red bars = species that increase both inside and outside of protected areas; blue = species that increase in protected areas but decrease outside of them; yellow = species that decrease in both protected areas and outside of them; grey = species that increase outside of protected areas but decline inside of them.

B. Results of analyses testing the effect of the year of protected area establishment (Supplementary Figures 4-5)

To evaluate the effect of variation in the year in which the protected area was established, we repeated the main analyses on a subset including only occurrence data starting from the year when the respective PA was established. This analysis was not performed for plants, due to the limited time span of these data (resulting in < 10 protected sites after subsetting).

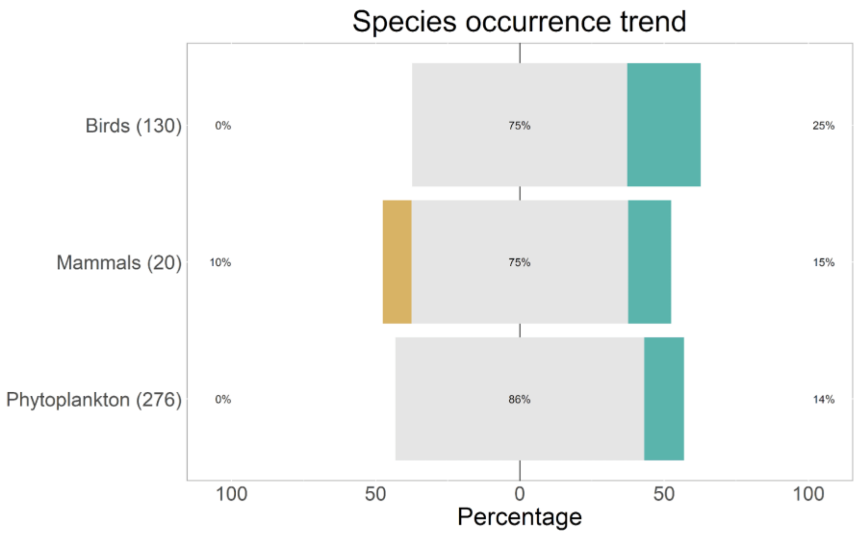

Supplementary Figure 4. Effect of variation to the timing of protection. The effectiveness of protected areas for the different taxonomic groups when years before the official protected area designation are excluded for each protected sites, and for the corresponding unprotected site. The figure shows the percentage of species with statistically significant support (defined with a 90% threshold) with a positive (green), negative (yellow), or no effect of protected areas on the occurrence trends of all species (total species number given within brackets).

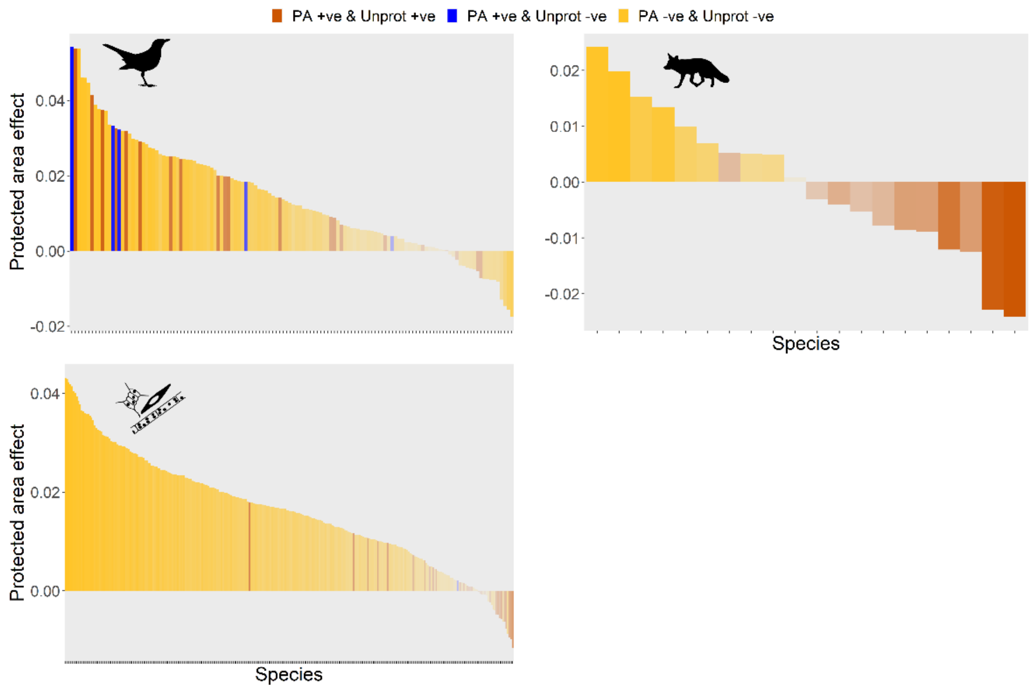

Supplementary Figure 5. Effect of variation in the timing of PA designation on species-level response. The species-level effectiveness of protected areas for the different taxonomic groups when years before the official protected area designation are excluded for each protected site, and for the corresponding unprotected sites. Values are derived from the effect size of the interaction term between protection and year in the joint species distribution models (to be compared with the results using all sites in Fig. 3 of the main manuscript). Bars represent the effect of protected areas on a given species, from the most positive (left) to the most negative (right). Colours indicate the level of statistical support: the darker the colour the higher is the statistical support for the effect on the species. Bar colors represent classes of species occurrence trends within and outside of protected areas: Red bars = species that increase both inside and outside of protected areas; blue = species that increase in protected areas but decrease outside of them; yellow = species that decrease in both protected areas and outside of them; grey = species that increase outside of protected areas but decline inside of them.

C. Results of analyses testing the effect of level of protection (IUCN protected area category; Supplementary Figures 6-7)

To evaluate the effect of variation in the level of protection (IUCN protected area category), we repeated the main analyses on a subset including only those sites whose protected area was classified at IUCN levels I to IV (that is, those with the strictest protection level), and their corresponding unprotected sites. This analysis was not performed for plants, due to the limited number of protected sites (n = 11) within categories I to IV for which plant data were available.

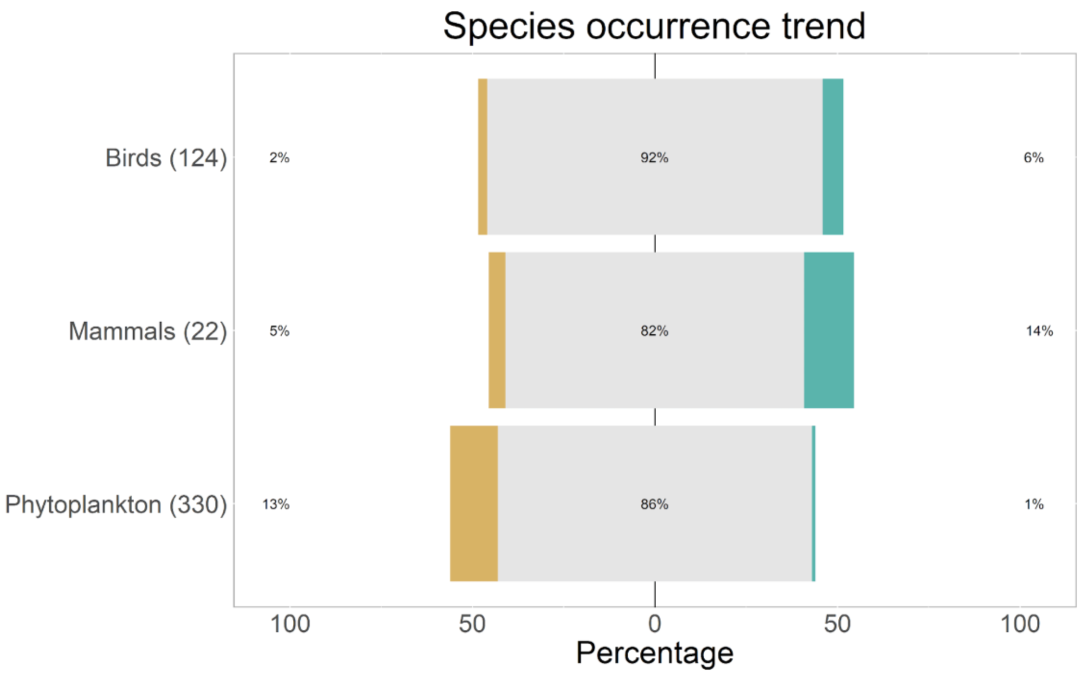

Supplementary Figure 6. Effect of variation in the protection level (IUCN category of protected areas). The effectiveness of protected areas for the different taxonomic groups when only sites with the strictest protection level (as classified at IUCN level I–IV) were included, along with their corresponding unprotected sites. The figure shows the percentage of species with statistically significant support (defined with a 90% threshold) with a positive (green), negative (yellow), or no effect of protected areas on the occurrence trends of all species (total species number given within brackets).

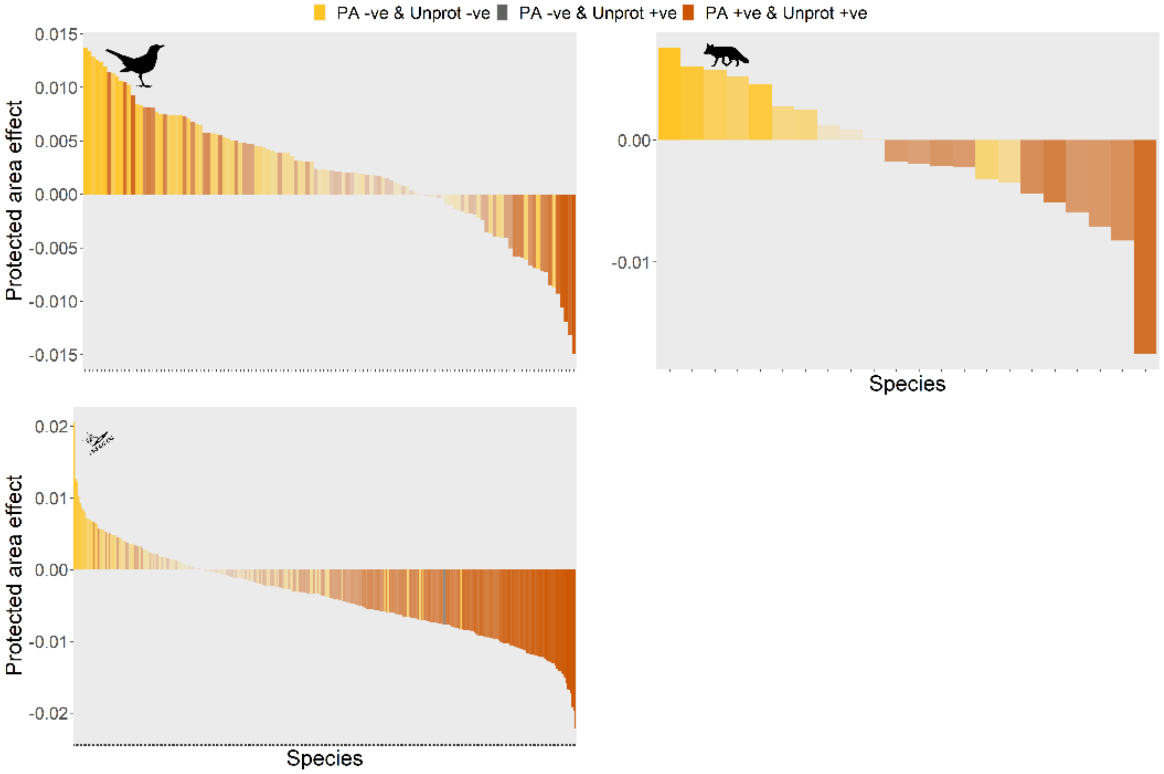

Supplementary Figure 7. Effect of variation in the protection level (IUCN category of protected areas) on species-level response. The species-level effectiveness of protected areas for the different taxonomic groups when only sites with the strictest protection level (as classified at IUCN level I–IV) were included, along with their corresponding unprotected sites. Values are derived from the effect size of the interaction term between protection and year in the joint species distribution models (to be compared with the results using all sites in Fig. 3 of the main manuscript). Bars represent the effect of protected areas on a given species, from the most positive (left) to the most negative (right). Colours indicate the level of statistical support: the darker the colour the higher is the statistical support for the effect on the species. Bar colors represent classes of species occurrence trends within and outside of protected areas: Red bars = species that increase both inside and outside of protected areas; blue = species that increase in protected areas but decrease outside of them; yellow = species that decrease in both protected areas and outside of them; grey = species that increase outside of protected areas but decline inside of them.

D. Sensitivity of results to the quantitative criteria used in assigning each site as protected versus unprotected (Supplementary Figures 8-11)

In assigning a site to being protected vs unprotected, we applied a buffer around the survey site. If the buffer intersected with a PA, the survey site was considered protected, otherwise unprotected. To evaluate the robustness of the results to these exact criteria, we repeated the main analyses on two subsets of the data: one from which the lowest quartile of buffer coverage by protected areas was excluded, and one from which the two lower quartiles of buffer coverage by protected areas were excluded.

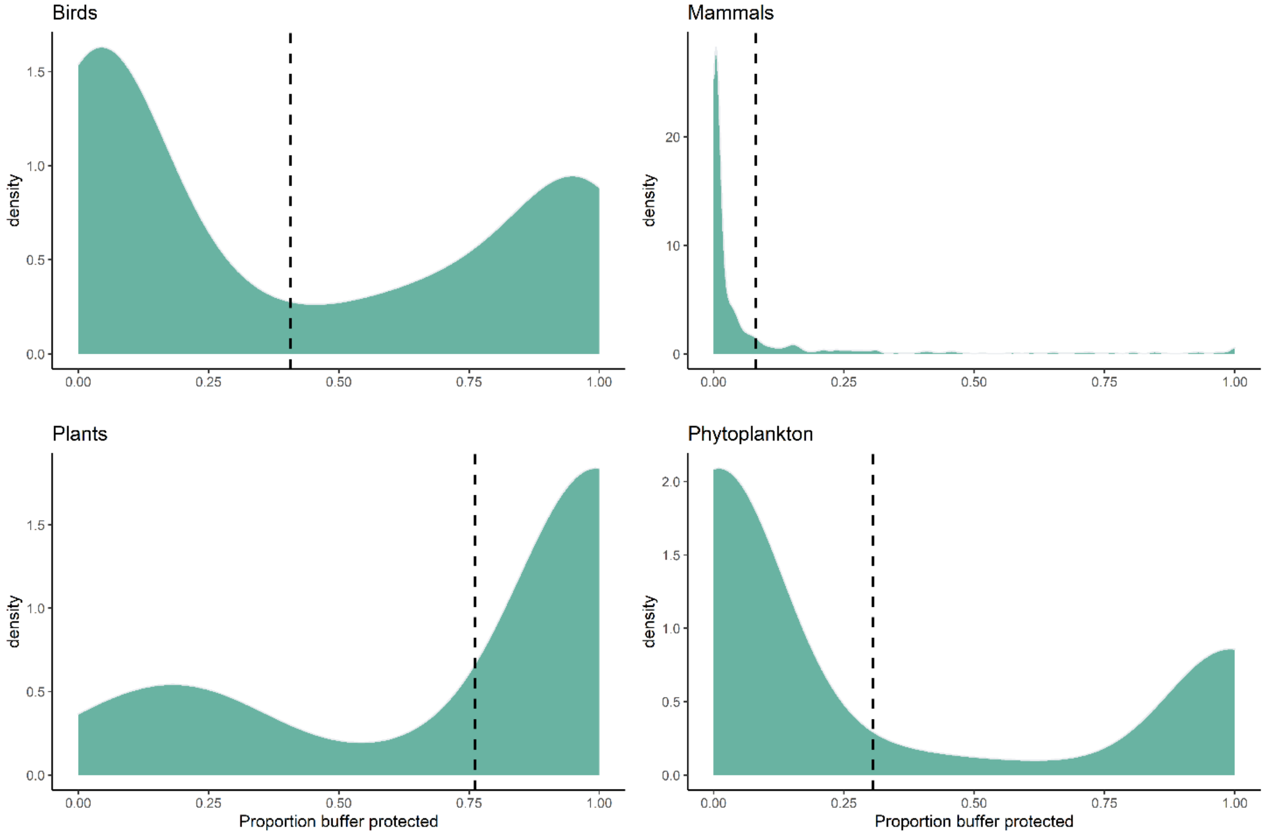

Supplementary Figure 8. Proportion of protected buffer for protected sites. Density plot showing the distribution of the proportion of protected area coverage for the buffers used to separate a site as protected versus unprotected. Here we show only the data relative to the sites classified as protected, i.e. those for which the buffer intercepts a protected area. Vertical dashed lines represent the mean value of the proportion of protected buffer for each taxonomic group.

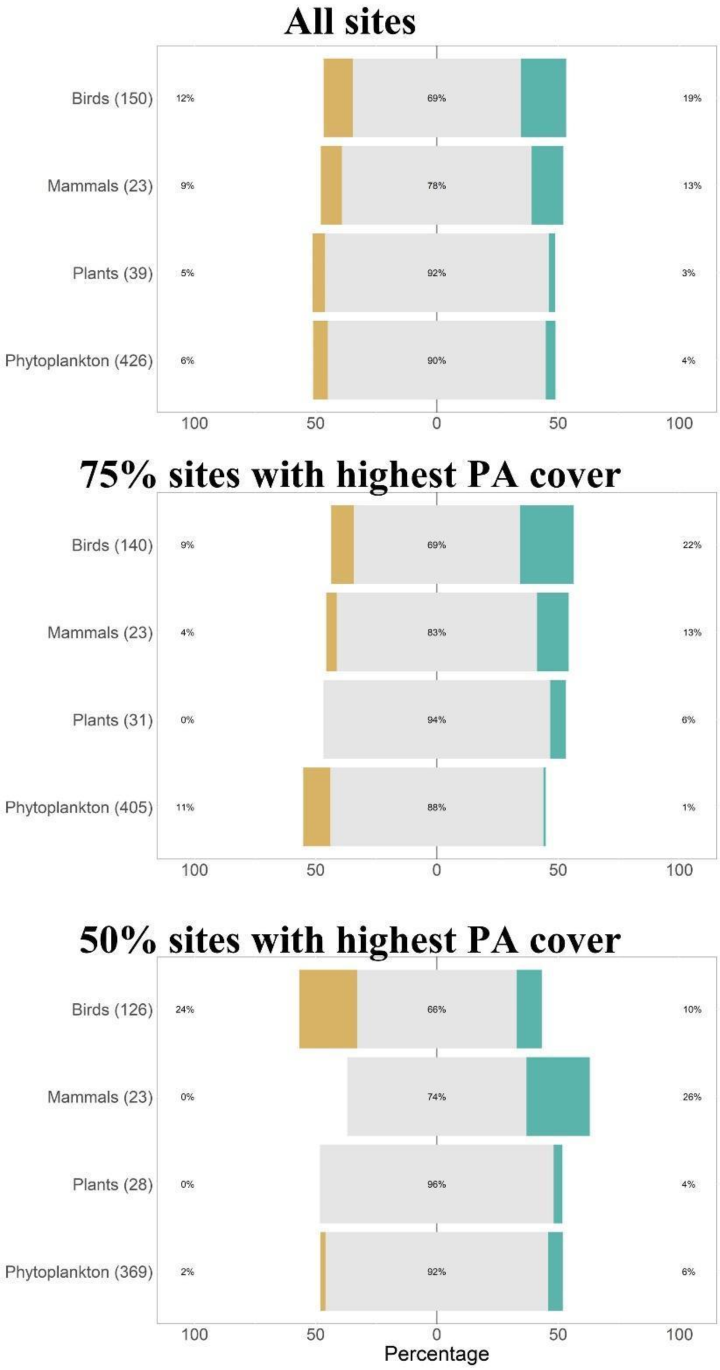

Supplementary Figure 9. Sensitivity of overall results to the protected area coverage. Overall results of the effectiveness of protected areas on the four taxonomic groups as it relates to the sensitivity to the proportion of the site coverage by protected areas, for the protected sites. The three panels show results when all data (as in Supplementary Figure 1 above) are considered (top panel), as well as results when the quartile with least coverage of protected areas is excluded (middle panel), and when the two quartiles with least coverage of protected areas are excluded (bottom panel). Each panel shows the percentage of species with statistically significant support (defined with a 90% threshold) with a positive (green), negative (yellow), or no effect of protected areas on the occurrence trends of all species (total species number given in within brackets).

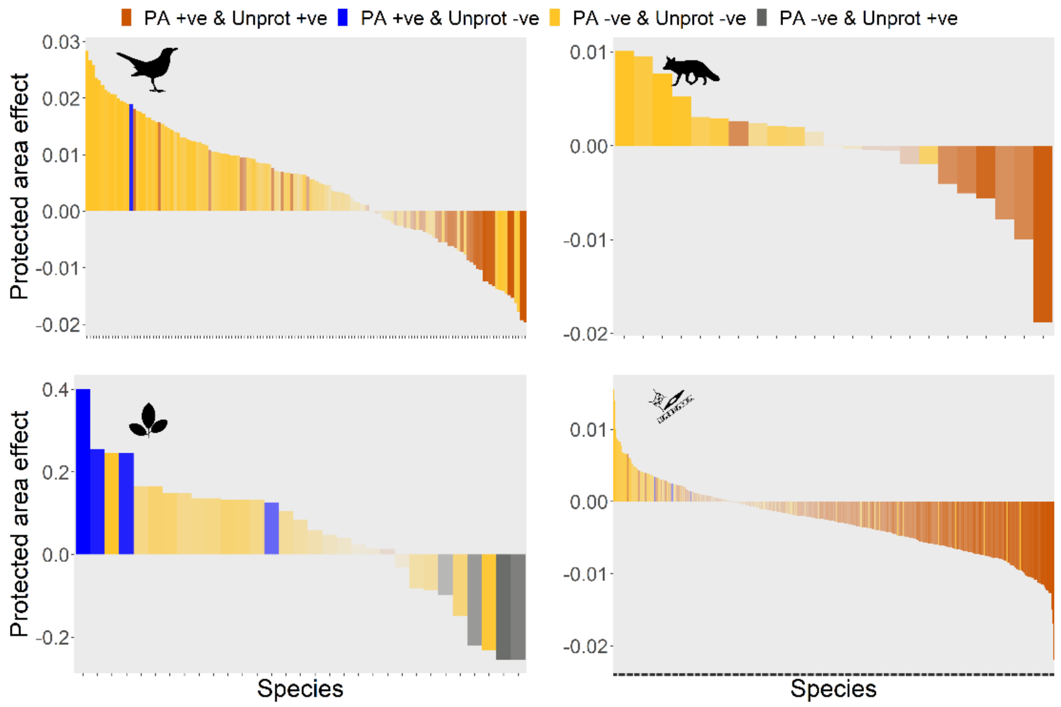

Supplementary Figure 10. Sensitivity of species-level results to the protected area coverage. The relative effect of the protected areas on occurrence at the species level when considering only the 75% of all sites with highest protected area coverage, and their corresponding unprotected sites. Values are as derived from the effect size of the interaction term between protection and year in the joint species distribution models (to be compared with the results using all sites in Fig. 3 of the main manuscript). Bars represent the effect of protected areas on a given species, from the most positive (left) to the most negative (right). The darker the colour the higher the statistical support for the effect on the species. Bar colors represent classes of species occurrence trends within and outside of protected areas: Red bars = species that increase both inside and outside of protected areas; blue = species that increase in protected areas but decrease outside; yellow = those who decrease in both protected areas and outside; grey = species that are increasing outside protected areas but declining inside.

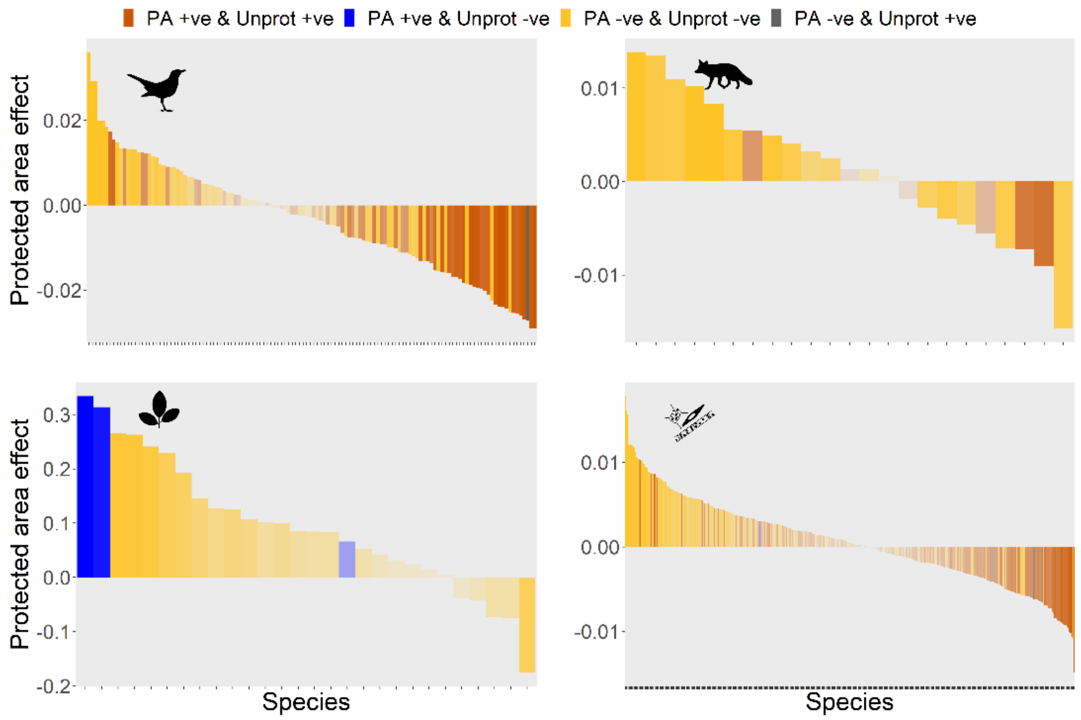

Supplementary Figure 11. Sensitivity of species-level results to the coverage of protected area. The relative effect of the protected areas on occurrence at the species level when considering only the 50% of all sites with highest protected area coverage, and their corresponding unprotected sites. Values are as derived from the effect size of the interaction term between protection and year in the joint species distribution models (to be compared with the results using all sites in Fig. 3 of the main manuscript). Bars represent the effect of protected areas on a given species, from the most positive (left) to the most negative (right). The darker the colour the higher the statistical support for the effect on the species. Bar colors represent classes of species occurrence trends within and outside of protected areas: Red bars = species that increase both inside and outside of protected areas; blue = species that increase in protected areas but decrease outside; yellow = those who decrease in both protected areas and outside; grey = species that are increasing outside protected areas but declining inside.

E. Sensitivity of results to latitudinal variation in PA designation (Supplementary Figures 12-13)

Sensitivity analyses quantifying the robustness of the results to the variation in the designation of PAs across Finland, with the largest PAs being located in the north (North Boreal zone), and the smaller sized PAs located towards the south of the country (see Fig. 1 in main text). We re-ran the main model on a subset of the data whereby the North Boreal zone sites are excluded, thereby verifying that the overall pattern holds when the region with the largest PAs is excluded.

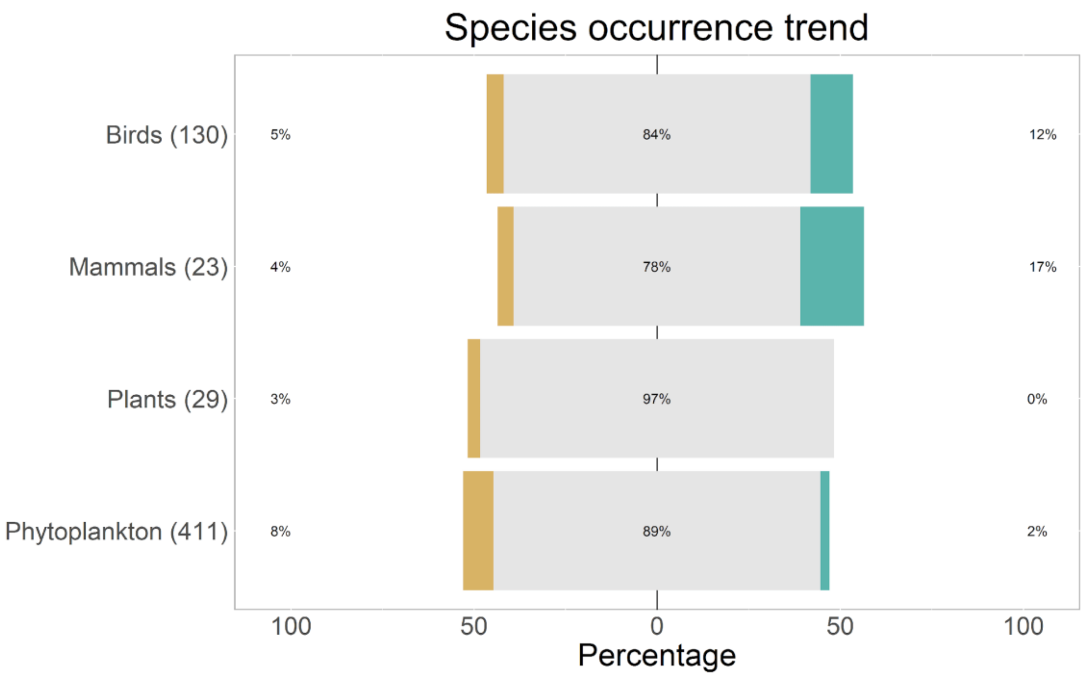

Supplementary Figure 12. Sensitivity of overall results to large PA extent in North Finland. Overall results of the effectiveness of protected areas on the four taxonomic groups when sites within the North Boreal zone, with highest cover of large protected areas and low human pressure, are excluded. The figure shows the percentage of species with statistically significant support (defined with a 90% threshold) with a positive (green), negative (yellow), or no effect of protected areas on the occurrence trends of all species (total species number given within brackets).

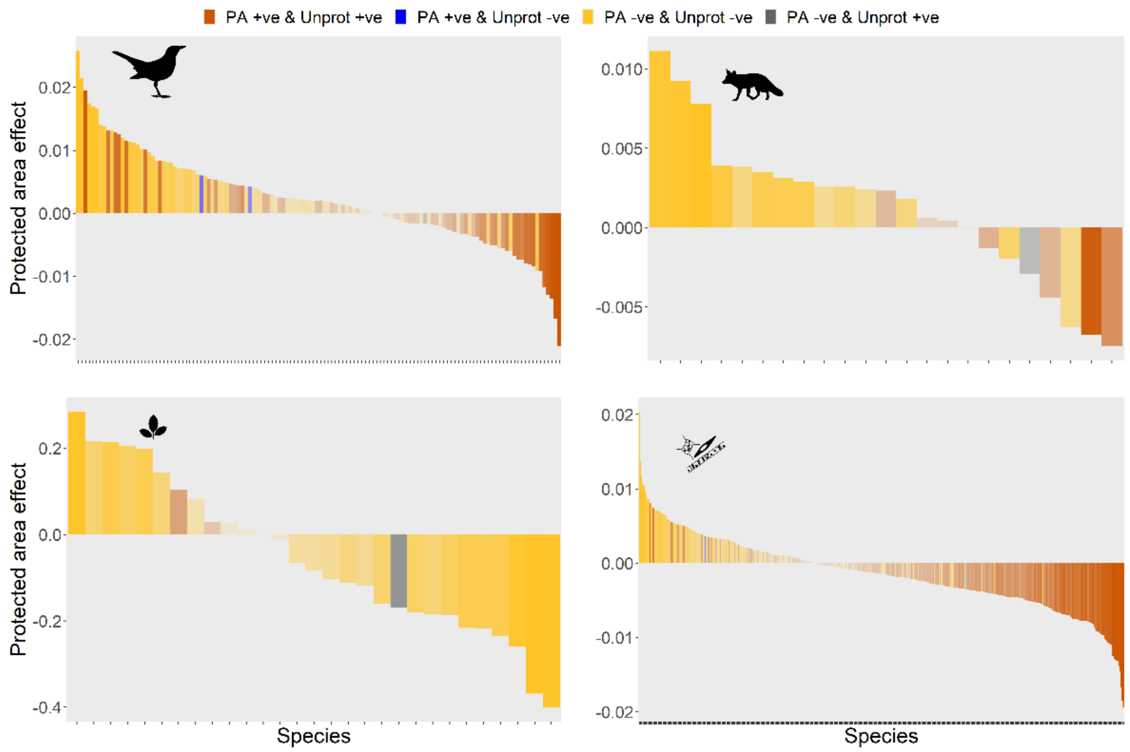

Supplementary Figure 13. Sensitivity of species-level results to large PA extent in North Finland. The relative effect of the protected areas on occurrence at the species level when sites within the North Boreal zone, with highest cover of large protected areas and low human pressure, are excluded. Values are as derived from the effect size of the interaction term between protection and year in the joint species distribution models (to be compared with the results using all sites in Fig. 3 of the main text). Bars represent the effect of protected areas on a given species, from the most positive (left) to the most negative (right). The darker the colour the higher the statistical support for the effect on the species. Bar colors represent classes of species occurrence trends within and outside of protected areas: Red bars = species that increase both inside and outside of protected areas; blue = species that increase in protected areas but decrease outside; yellow = those who decrease in both protected areas and outside; grey = species that are increasing outside protected areas but declining inside.

F. Performance results and comparison of alternative methods for matching protected and unprotected sites (Supplementary Figures 14-21 show results per taxonomic group and matching method).

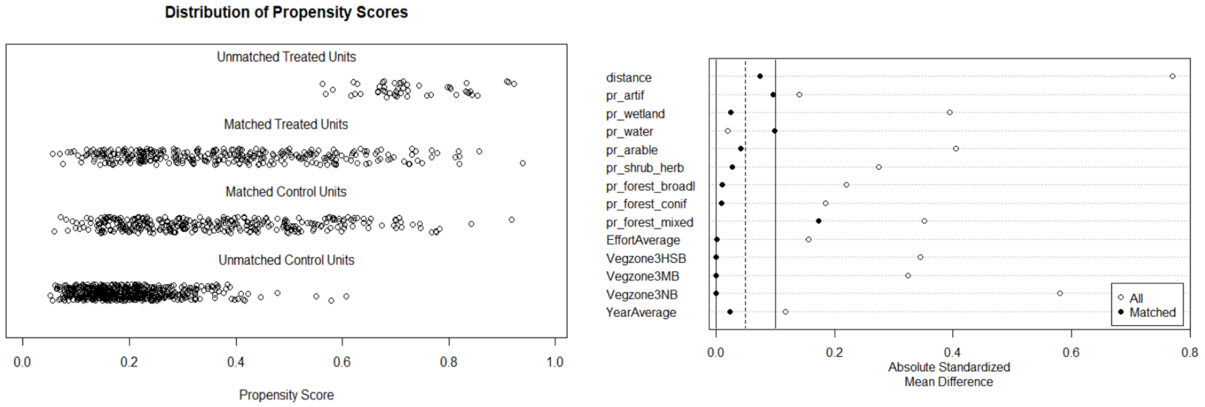

Supplementary Figure 14. Mahalanobis distance method performance on the bird data. The left panel shows the overall propensity score distributions across all variables used for matching for the treatment (protected area) and the control (unprotected) units (sites), separately for the unmatched (i.e. before matching) and matched (after matching) units. The right panel shows the absolute standardized mean difference (between protected and unprotected sites) as one value for each of the covariates used for matching (listed in the Y axis). Empty circles depict the difference between all protected and unprotected sites before matching, and black filled circles depict the difference between matched sites. Low values close to zero indicate low differences between protected and unprotected sites for a given covariate.

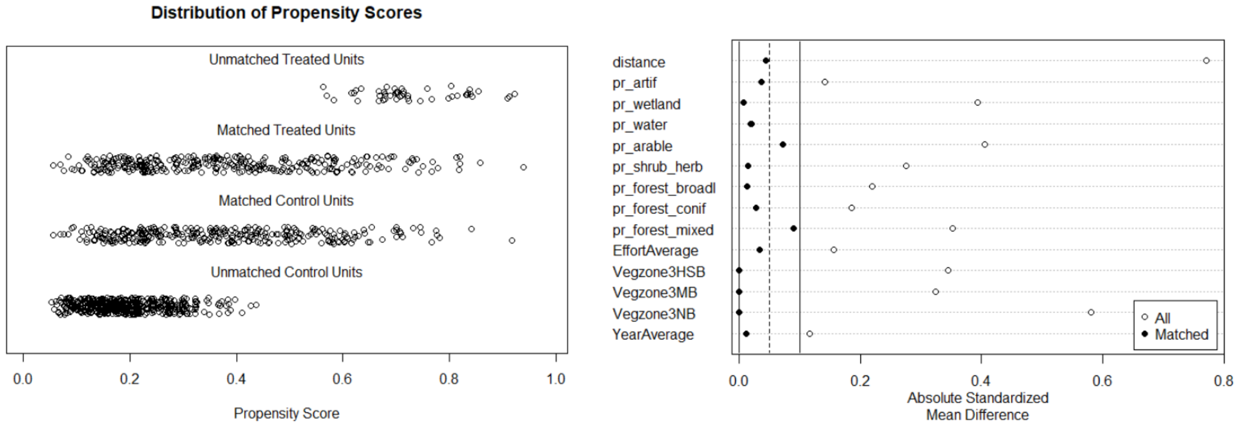

Supplementary Figure 15. Nearest neighbor propensity score matching method performance on the bird data. The left panel shows the overall propensity scores distribution across all variables used for matching for the treatment (protected area) and the control (unprotected) units (sites), separately for the unmatched (i.e. before matching) and matched (after matching) units. The right panel shows the absolute standardized mean difference (between protected and unprotected sites) as one value for each of the covariates used for matching (listed in the Y axis). Empty circles depict the difference between all protected and unprotected sites before matching, and black filled circles depict the difference between matched sites.

Low values close to zero indicate low differences between protected and unprotected sites for a given covariate.

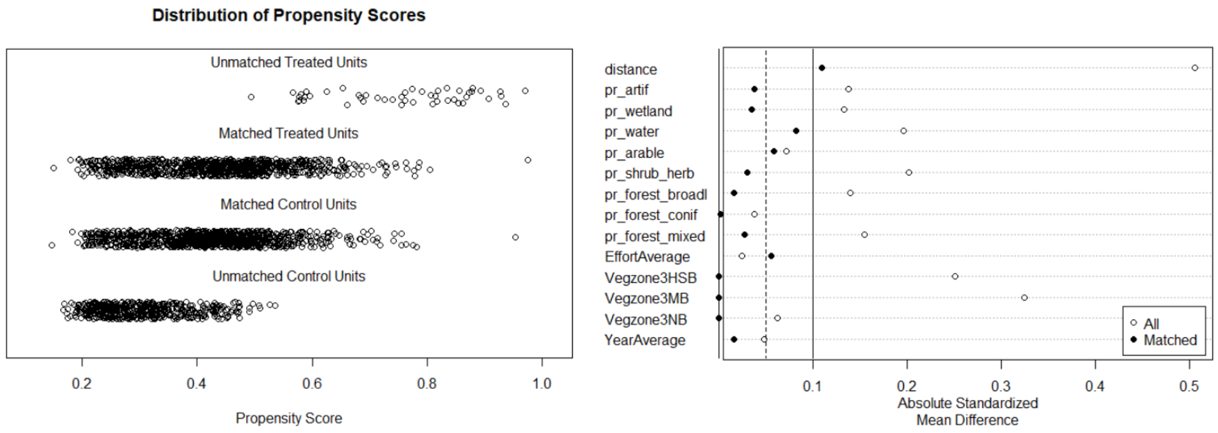

Supplementary Figure 16. Mahalanobis distance method performance on the mammal data. The left panel shows the overall propensity scores distribution across all variables used for matching for the treatment (protected area) and the control (unprotected) units (sites) for the unmatched (i.e. before matching) and matched (after matching) units. The right panel shows the absolute standardised mean difference (between protected and unprotected sites) as one value for each of the covariates used for matching (listed in the Y axis). Empty circles depict the difference between all protected and unprotected sites before matching, and black filled circles depict the different between matched sites. Low values close to zero indicate low differences between protected and unprotected sites for a given covariate.

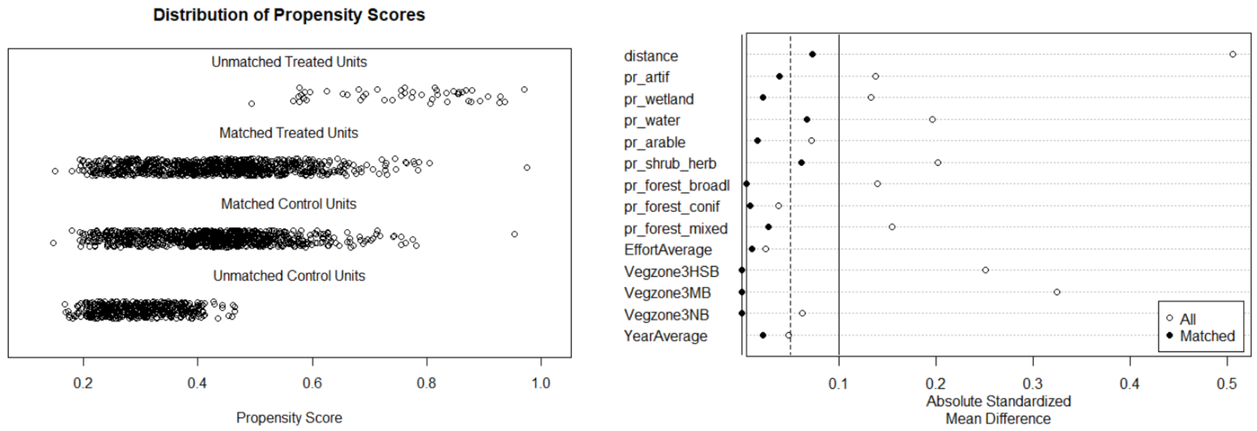

Supplementary Figure 17. Nearest neighbor propensity score matching method performance on the mammal data. The left figure shows the overall propensity scores distribution across all variables used for matching for the treatment (protected area) and the control (unprotected) units (sites) for the unmatched (i.e. before matching) and matched (after matching) units. The right figure shows the absolute standardised mean difference (between protected and unprotected sites) as one value for each of the covariates used for matching (listed in the Y axis). Empty circles depict the difference between all protected and unprotected sites before matching, and black filled circles depict the different between matched sites. Low values close to zero indicate low differences between protected and unprotected sites for a given covariate.

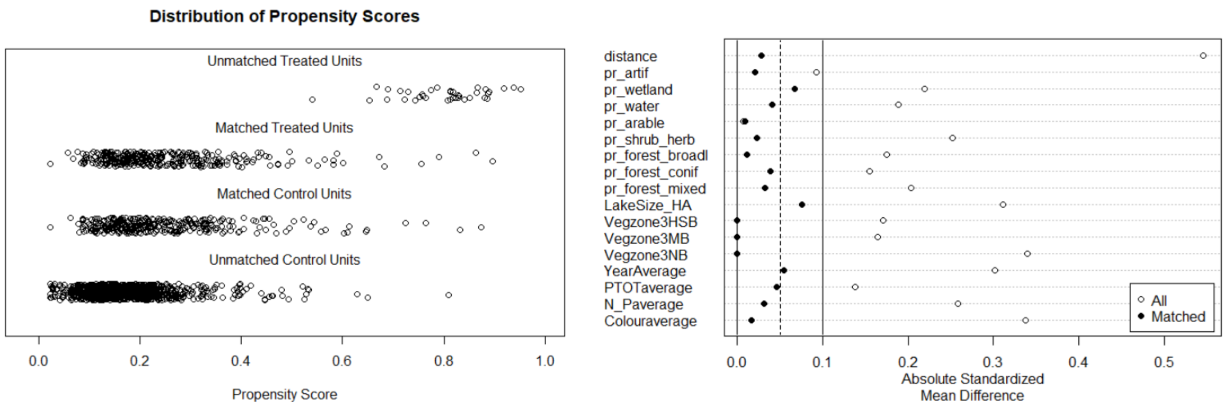

Supplementary Figure 18. Mahalanobis distance method performance on the phytoplankton data. The left figure shows the overall propensity scores distribution across all variables used for matching for the treatment (protected area) and the control (unprotected) units (sites) for the unmatched (i.e. before matching) and matched (after matching) units. The right figure shows the absolute standardised mean difference (between protected and unprotected sites) as one value for each of the covariates used for matching (listed in the Y axis). Empty circles depict the difference between all protected and unprotected sites before matching, and black filled circles depict the difference between matched sites. Low values close to zero indicate low differences between protected and unprotected sites for a given covariate.

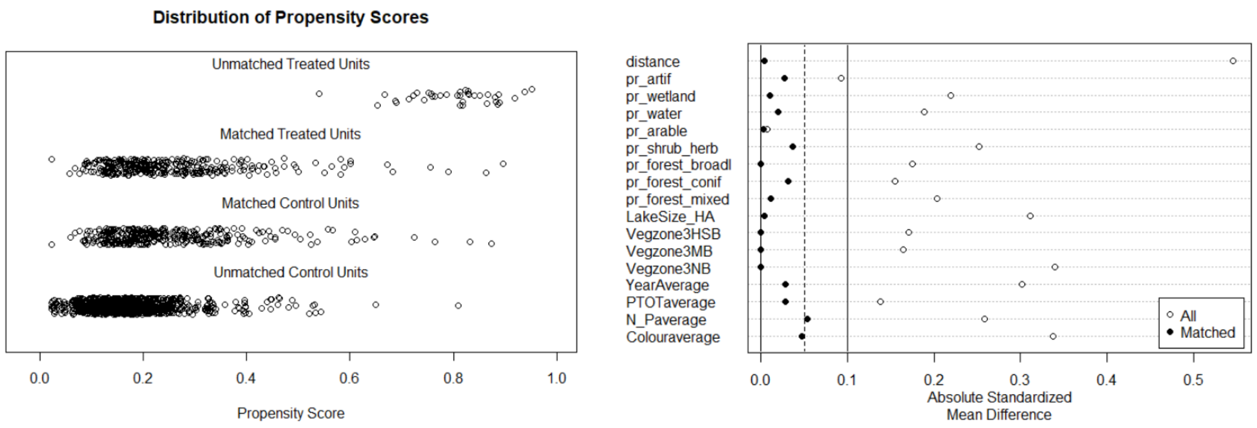

Supplementary Figure 19. Nearest neighbor propensity score matching method performance on the phytoplankton data. The left figure shows the overall propensity scores distribution across all variables used for matching for the treatment (protected area) and the control (unprotected) units (sites) for the unmatched (i.e. before matching) and matched (after matching) units. The right figure shows the absolute standardised mean difference (between protected and unprotected sites) as one value for each of the covariates used for matching (listed in the Y axis). Empty circles depict the difference between all protected and unprotected sites before matching, and black filled circles depict the difference between matched sites. Low values close to zero indicate low differences between protected and unprotected sites for a given covariate.

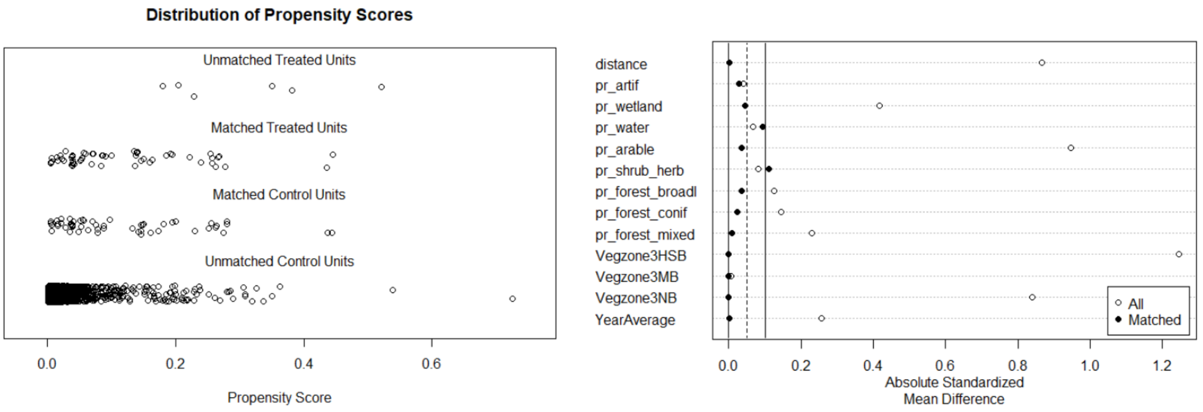

Supplementary Figure 20. Mahalanobis distance method performance on the plant data. The left figure shows the overall propensity scores distribution across all variables used for matching for the treatment (protected area) and the control (unprotected) units (sites) for the unmatched (i.e. before matching) and matched (after matching) units. The right figure shows the absolute standardised mean difference (between protected and unprotected sites) as one value for each of the covariates used for matching (listed in the Y axis). Empty circles depict the difference between all protected and unprotected sites before matching, and black filled circles depict the difference between matched sites. Low values close to zero indicate low differences between protected and unprotected sites for a given covariate.

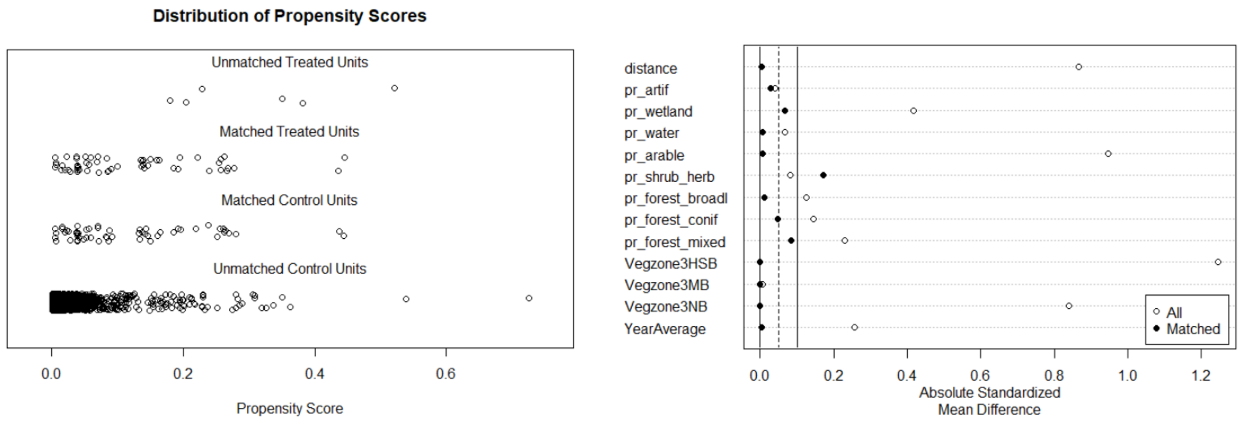

Supplementary Figure 21. Nearest neighbor propensity score matching method performance on the plant data. The left figure shows the overall propensity scores distribution across all variables used for matching for the treatment (protected area) and the control (unprotected) units (sites) for the unmatched (i.e. before matching) and matched (after matching) units. The right figure shows the absolute standardised mean difference (between protected and unprotected sites) as one value for each of the covariates used for matching (listed in the Y axis). Empty circles depict the difference between all protected and unprotected sites before matching, and black filled circles depict the difference between matched sites. Low values close to zero indicate low differences between protected and unprotected sites for a given covariate.

G. Checking for phylogenetic signal in the species level response to protection (Supplementary Figures 22-25):

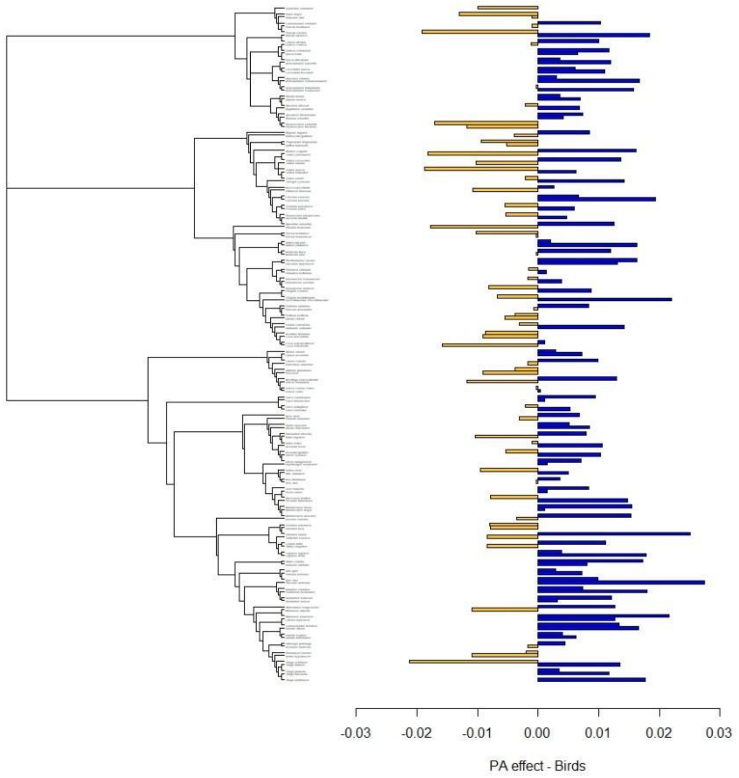

Supplementary Figure 22. The species level effect of protected areas across the phylogeny of birds. The bird tree is shown on the left and was based on a consensus across 100 Ericson trees (downloaded from vertlife.org). The protected area effect for each species, from negative (yellow) to positive (blue) is shown on the right panel, whose values are derived from the main model (results presented in Figure 2 of the main manuscript). The effect of protected areas on birds is not phylogenetically structured ($\lambda = 0.013$, $K = 0.026$, both with $p > 0.05$; based on 2-sample T-test of significance).

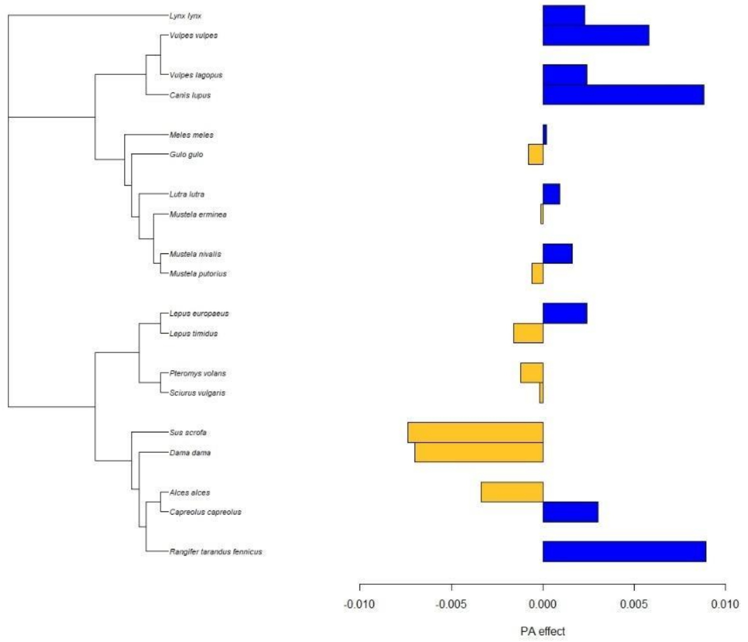

Supplementary Figure 23. The species level effect of protected areas across the phylogeny of mammals. The mammal tree is shown on the left and based on a consensus across 100 trees (downloaded from vertlife.org). The protected area effect for each species, from negative (yellow) to positive (blue) is shown on the right panel, the values of which are derived from the main model (results presented in Figure 2 of the main manuscript). The effect of protected areas on mammals is not phylogenetically structured ($\lambda < 0.001$, $K = 0.224$, both with $p > 0.05$; based on 2-sample T-test of significance).

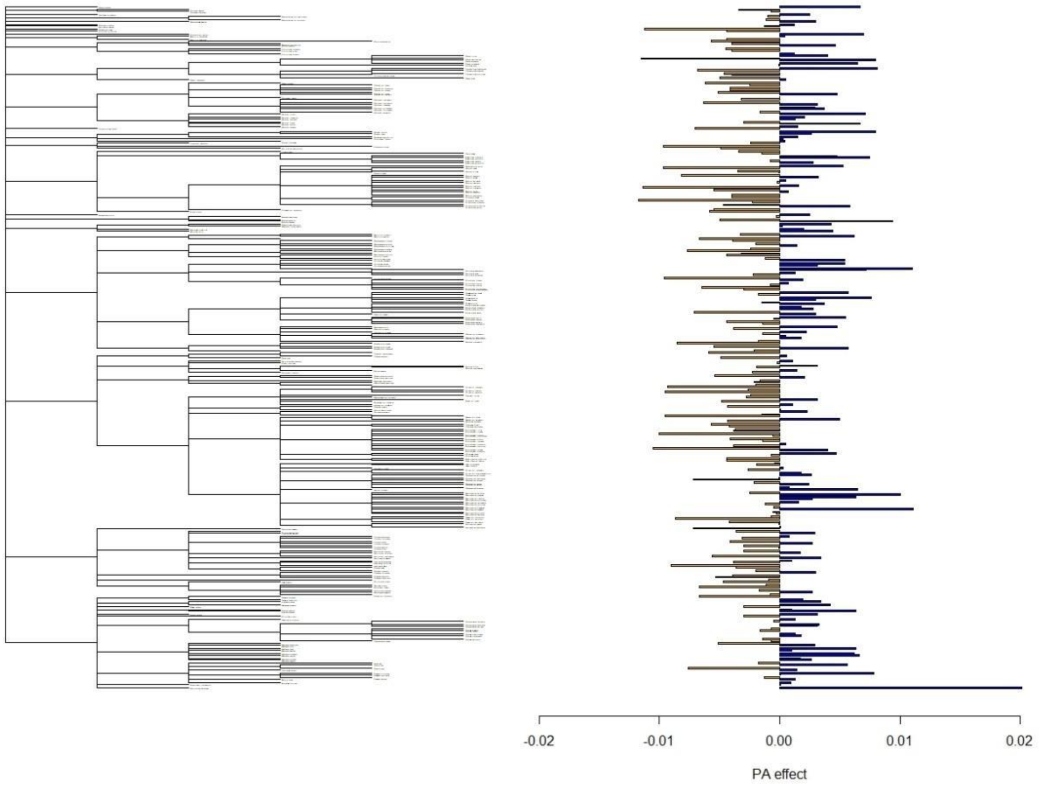

Supplementary Figure 24. The species level effect of protected areas across the phylogeny of phytoplankton. The phytoplankton tree is derived using the relative group taxonomy and assuming an equal branch length across the tree, as no phylogeny is available for this group. The protected area effect for each species, from negative (yellow) to positive (blue) is shown on the right panel, whose values are derived from the main model (results presented in Figure 2 of the main manuscript). The effect of protected areas on phytoplankton is weakly phylogenetically structured ($\lambda = 0.332$ with $p = 0.0005$, but $K = 0.36$ and $p = 0.18$; based on 2-sample T-test of significance). The highly significant p-value for the λ may be more related to the high number of species, than to a genuine phylogenetic structure of the effect of protected areas, which is weak based on the λ value[1].

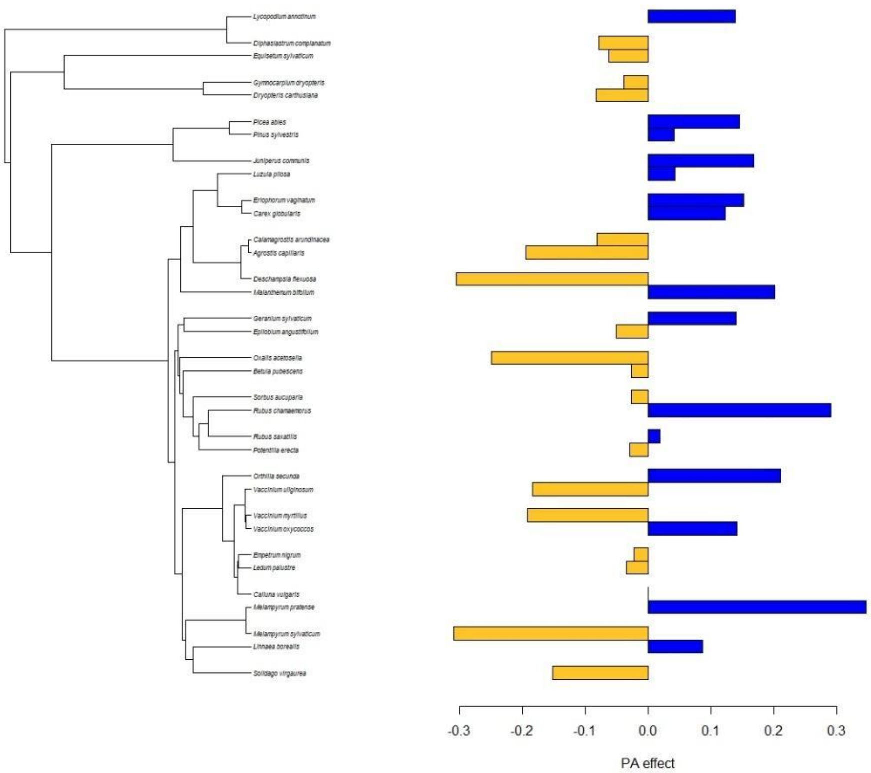

Supplementary Figure 25. The species level effect of protected areas across the phylogeny of plants. The plant tree is shown on the left and based on available plant phylogeny[2,3]. The protected area effect for each species, from negative (yellow) to positive (blue) is shown on the right panel, whose values are derived from the main model (results presented in Figure 2 of the main manuscript). The effect of protected areas on plants is not phylogenetically structured ($\lambda < 0.001$, $K = 0.066$, both with $p > 0.05$; based on 2-sample T-test of significance).

References for supplementary material:

1 Collyer, M. L., Baken, E. K., & Adams, D. C. (2022). A standardized effect size for evaluating and comparing the strength of phylogenetic signal. *Methods in Ecology and Evolution*, *13*(2), 367-382.

2 Smith, S. A., & Brown, J. W. (2018). Constructing a broadly inclusive seed plant phylogeny. *American journal of botany*, *105*(3), 302-314.

3 Zanne, A. E., Tank, D. C., Cornwell, W. K., Eastman, J. M., Smith, S. A., FitzJohn, R. G., ... & Beaulieu, J. M. (2014). Three keys to the radiation of angiosperms into freezing environments. *Nature*, *506*(7486), 89-92.
