# Supplementary material for: Mixed effects of a national protected area network on terrestrial and freshwater biodiversity
Source: Nat Commun. 2023 Sep 13;14:5426. doi: 10.1038/s41467-023-41073-4 (PMC10499833; doi:10.1038/s41467-023-41073-4)
Supplement: Supplementary file 3 — Reporting Summary [file 41467_2023_41073_MOESM3_ESM.pdf]

nature portfolio

Corresponding author(s): Andrea Santangeli
Last updated by author(s): Aug 21, 2023

Reporting Summary

Nature Portfolio wishes to improve the reproducibility of the work that we publish. This form provides structure for consistency and transparency in reporting. For further information on Nature Portfolio policies, see our Editorial Policies and the Editorial Policy Checklist.

Statistics

For all statistical analyses, confirm that the following items are present in the figure legend, table legend, main text, or Methods section.

n/a	Confirmed	
☐	☒	The exact sample size (*n*) for each experimental group/condition, given as a discrete number and unit of measurement
☐	☒	A statement on whether measurements were taken from distinct samples or whether the same sample was measured repeatedly
☐	☒	The statistical test(s) used AND whether they are one- or two-sided<br>*Only common tests should be described solely by name; describe more complex techniques in the Methods section.*
☐	☒	A description of all covariates tested
☐	☒	A description of any assumptions or corrections, such as tests of normality and adjustment for multiple comparisons
☐	☒	A full description of the statistical parameters including central tendency (e.g. means) or other basic estimates (e.g. regression coefficient) AND variation (e.g. standard deviation) or associated estimates of uncertainty (e.g. confidence intervals)
☐	☒	For null hypothesis testing, the test statistic (e.g. *F*, *t*, *r*) with confidence intervals, effect sizes, degrees of freedom and *P* value noted<br>*Give P values as exact values whenever suitable.*
☐	☒	For Bayesian analysis, information on the choice of priors and Markov chain Monte Carlo settings
☒	☐	For hierarchical and complex designs, identification of the appropriate level for tests and full reporting of outcomes
☐	☒	Estimates of effect sizes (e.g. Cohen's *d*, Pearson's *r*), indicating how they were calculated

Our web collection on statistics for biologists contains articles on many of the points above.

Software and code

Policy information about availability of computer code

Data collection	No specific software was used for data collection.
Data analysis	All analyses have been conducted in R software (version 4.2.0), using primarily the MatchIt package (version 4.5.0) for matching protected and unprotected sites, and Hmsc package (version 3.0-13) for the joint species distribution modeling. The code to replicate the analyses is stored in Figshare at the following link: https://figshare.com/s/fcdd09b906d03abc0ac7

For manuscripts utilizing custom algorithms or software that are central to the research but not yet described in published literature, software must be made available to editors and reviewers. We strongly encourage code deposition in a community repository (e.g. GitHub). See the Nature Portfolio guidelines for submitting code & software for further information.

Data

Policy information about availability of data

All manuscripts must include a data availability statement. This statement should provide the following information, where applicable:

- Accession codes, unique identifiers, or web links for publicly available datasets
- A description of any restrictions on data availability
- For clinical datasets or third party data, please ensure that the statement adheres to our policy

All the wildlife occurrence data used for the analyses are available in an online repository at Figshare at the following link: https://figshare.com/s/

fcdd09b906d03abc0ac7. The raw wildlife observation data, original data before filtering, are available from different sources and with different conditions. Bird data are available through the Finnish Biodiversity Information Facility, FinBIF (https://laji.fi/en/). Mammal and plant data are available upon request from the Natural Resources Institute (LUKE - requests should be sent to kirjaamo@luke.fi). Phytoplankton data are available through the open access data service of Finnish Environment Institute (SYKE). Spatial data from the WDPA World Database on Protected Areas (accession time: January 2021) are freely available at www.protectedplanet.net. The land-use variables (accessed on January 2021) are freely available from this link: https://land.copernicus.eu/pan-european/corine-land-cover/clc-2000.

Human research participants

Policy information about studies involving human research participants and Sex and Gender in Research.

Reporting on sex and gender	Not applicable
Population characteristics	Not applicable
Recruitment	Not applicable
Ethics oversight	Not applicable

Note that full information on the approval of the study protocol must also be provided in the manuscript.

Field-specific reporting

Please select the one below that is the best fit for your research. If you are not sure, read the appropriate sections before making your selection.

☐ Life sciences ☐ Behavioural & social sciences ☒ Ecological, evolutionary & environmental sciences

For a reference copy of the document with all sections, see nature.com/documents/nr-reporting-summary-flat.pdf

Ecological, evolutionary & environmental sciences study design

All studies must disclose on these points even when the disclosure is negative.

Study description	We used a robust matching to obtain a comparable set of protected and unprotected wildlife survey sites, and then employed a joint species distribution modeling on patterns of occurrence of over 600 species between birds, mammals, plants and phytoplankton across Finland.
Research sample	We used four decades of systematic high-resolution wildlife monitoring data from Finland, including over 600 different species from across a latitudinal gradient of over 1000km.
Sampling strategy	Each taxon-specific dataset employs a specific sampling method. No sampling strategy was implemented as part of this study
Data collection	We analyzed previously collected large data provided by several collaborators at key institutions in Finland. The key person in charge of each data collection/curation was offered co-authorship on this study.
Timing and spatial scale	Data span across the past four decades, with some variation between the different taxa-specific datasets, with data collected across most of the terrestrial and freshwater area of Finland.
Data exclusions	Species with a minimum of 10 occurrences across the whole taxon-specific dataset were included for the joint species distribution modeling. Protected area sites and those unprotected that did not fit the stringent matching criteria, aimed to achieve as similar protected and unprotected sites, have been excluded.
Reproducibility	The results are reproducible by using the occurrence data and the available R code.
Randomization	No randomization procedure was applicable for this study
Blinding	Blinding is not relevant for this study

Did the study involve field work? ☐ Yes ☒ No

Reporting for specific materials, systems and methods

We require information from authors about some types of materials, experimental systems and methods used in many studies. Here, indicate whether each material, system or method listed is relevant to your study. If you are not sure if a list item applies to your research, read the appropriate section before selecting a response.

Materials & experimental systems

n/a	Involved in the study	
☒	☐	Antibodies
☒	☐	Eukaryotic cell lines
☒	☐	Palaeontology and archaeology
☒	☐	Animals and other organisms
☒	☐	Clinical data
☒	☐	Dual use research of concern

Methods

n/a	Involved in the study	
☒	☐	ChIP-seq
☒	☐	Flow cytometry
☒	☐	MRI-based neuroimaging
