# Supplementary material for: Mixed effects of a national protected area network on terrestrial and freshwater biodiversity
Source: Nat Commun. 2023 Sep 13;14:5426. doi: 10.1038/s41467-023-41073-4 (PMC10499833; doi:10.1038/s41467-023-41073-4)
Supplement: Supplementary file 5 — Description of Additional Supplementary Files [file 41467_2023_41073_MOESM5_ESM.pdf]

File Name: Supplementary Data 1

Description: Species level estimates from the model presented in the main text showing, for each species within each of the four taxonomic groups, the mean effect as well as statistical support relative to the interaction term *protection* (protected vs unprotected) * *year* tested in the main model. The direction of the effect variable indicates whether there was a significantly supported (defined with a 90% support threshold) effect of protection, either positive or negative, or if there was no statistically supported effect (indicated as “stable” in the table). The data are presented as an excel spreadsheet available online.
